# Concurrent versus sequential immune checkpoint inhibition in the neoadjuvant treatment of resectable esophageal squamous cell carcinoma: a systematic review and meta-analysis

**DOI:** 10.1016/j.ctro.2026.101289

**Published:** 2026-09-10

**Authors:** Reza Ghalehtaki, Alireza Shariati, Taher Fanihagh, Zahra Moradi, Niloofar Moradi

**Affiliations:** a Department of Radiation Oncology, Cancer Institute, School of Medicine, Tehran University of Medical Sciences, Tehran, Iran; b Radiation Oncology Research Center, Cancer Research Institute, Tehran University of Medical Sciences, Tehran, Iran; c School of Medicine, Tehran University of Medical Sciences, Tehran, Iran

**Keywords:** Esophageal squamous cell carcinoma, Neoadjuvant chemoradiotherapy, Immune checkpoint inhibitors, Treatment sequencing, Pathological complete response, Major pathological response, Systematic review and meta-analysis

## Abstract

**Background:**

Immune checkpoint inhibitors (ICIs) are increasingly integrated into the neoadjuvant treatment of resectable esophageal squamous cell carcinoma (ESCC), yet the optimal timing of ICI administration remains undefined. We conducted the first systematic review and meta-analysis directly comparing these two strategies.

**Methods:**

PubMed, Embase, and Scopus were searched from inception through April 2026. Prospective studies enrolling adult patients with non-metastatic resectable ESCC who received neoadjuvant ICI alongside chemotherapy or chemoradiotherapy were eligible. Pooled pathological complete response (pCR) and major pathological response (MPR) were analyzed using random-effects models.

**Results:**

Fifty-three prospective studies (2937 patients) were included. In the primary analysis, consolidation ICI was associated with significantly higher pCR than concurrent ICI (48% vs. 31%; *p* = 0.0005). However, when restricted to studies using chemoradiotherapy in both arms, this difference lost statistical significance (48% vs. 41%; *p* = 0.18), suggesting the initial advantage was largely attributable to a greater use of chemoradiotherapy in the sequential subgroup rather than ICI timing per se. A parallel pattern was observed for MPR.

**Conclusions:**

When matched for treatment backbone, concurrent and consolidation ICI timing produce comparable pathological response rates in resectable ESCC. Randomized trials with pre-specified timing arms and biomarker stratification are warranted.

## Highlights

• This is the first systematic review and meta-analysis comparing concurrent versus sequential ICI timing in neoadjuvant treatment of resectable ESCC.
• In the primary analysis, sequential (consolidation) ICI was associated with significantly higher pCR and MPR rates than concurrent ICI.
• When analyses were restricted to studies with chemoradiotherapy in both arms, the differences in pCR and MPR were no longer statistically significant.
• The apparent advantage of sequential ICI was largely driven by differences in treatment backbone rather than ICI timing itself.
• Concurrent and sequential ICI may provide comparable pathological responses when combined with similar neoadjuvant regimens in resectable ESCC.

## 1 Introduction

Esophageal cancer ranks among the most lethal malignancies worldwide. According to GLOBOCAN 2022 estimates, approximately 511,000 new cases were diagnosed globally, resulting in over 445,000 deaths, with roughly 75% of cases and deaths occurring in Asia [1]. Projections suggest the global burden will approach close to one million cases annually by 2040. Of the two major histological subtypes, ESCC accounts for approximately 90% of all cases, with the highest incidence in Eastern Asia and Southern Africa. [1], [2].

For patients with resectable locally advanced disease, multimodal therapy combining neoadjuvant treatment followed by surgery has become the standard approach. The CROSS trial established neoadjuvant chemoradiotherapy followed by esophagectomy as a benchmark, reporting a median survival of 81.6 months in the neoadjuvant chemoradiotherapy plus surgery group versus 21.1 months in the surgery alone group, for the ESCC subgroup specifically [3], [4]. Despite these gains, the majority of patients still experience disease recurrence, highlighting a clear ceiling effect of conventional neoadjuvant chemoradiotherapy and the need for more effective strategies [5].

Immune checkpoint inhibitors (ICIs) targeting the PD-1/PD-L1 axis have transformed the management of advanced ESCC. Multiple phase III trials have established ICI-based combination therapy as the standard first-line treatment for advanced ESCC. The KEYNOTE-590, ESCORT-1st, CheckMate-648, JUPITER-06, ORIENT-15, and RATIONALE-306 trials all demonstrated significantly improved overall survival with the addition of PD-1 inhibitors to chemotherapy compared to chemotherapy alone [6], [7], [8], [9], [10], [11].

This success has prompted growing interest in integrating ICIs into the neoadjuvant setting, mostly as immune-chemotherapy (ICT) or immune-chemoradiotherapy (ICRT). The phase III ESCORT-NEO trial demonstrated that adding camrelizumab concurrently to neoadjuvant chemotherapy significantly improved pCR rates compared with chemotherapy alone (28.0% in the camrelizumab plus albumin-bound paclitaxel and cisplatin arm versus 4.7% in the chemotherapy-alone arm) [12]. A subsequent meta-analysis confirmed that neoadjuvant ICI combined with chemotherapy was associated with significantly higher pCR and MPR rates relative to chemotherapy alone, without significant differences in treatment-related adverse events or postoperative complications [13].

When combined with chemoradiotherapy, pooled pCR rates appear substantially higher (approximately 48% for neoadjuvant ICI plus chemoradiotherapy versus 29% for neoadjuvant ICI plus chemotherapy), though at the cost of higher grade 3–4 adverse event rates [14].

A critical unresolved question however, is the optimal timing of ICI administration relative to chemotherapy or chemoradiotherapy. ICIs can be delivered concurrently alongside the cytotoxic backbone, or sequentially, either before or after the chemoradiotherapy phase. These schedules carry potentially distinct immunological, efficacy, and toxicity profiles. When administered concurrently, ICIs can exploit radiation-induced PD-L1 upregulation and the immediate release of tumor antigens and damage-associated molecular patterns (DAMPs), theoretically maximizing the immunogenic priming window while the antigen-presenting machinery is most active [15]. However, chemoradiotherapy simultaneously depletes circulating lymphocytes particularly those reside within or in the nearby lymph nodes to the irradiated target volume. These T-lymphocytes are the very effector cells that ICIs depend upon. However, they are affected through direct radiation-induced lymphopenia given their high radiosensitivity even at low doses of radiation [16]. This creates a biological paradox as the concurrent approach may trigger immune activation while simultaneously impairing the cellular machinery needed to execute it. By contrast, sequential administration may allow immunological changes induced by chemoradiotherapy to mature while the body recovers from the immune-depletion state. This potentially preserves the efficacy while reducing cumulative toxicity before ICI exposure [17], [18]. Indirect evidence from non-small cell lung cancer (NSCLC) suggests a similar pattern, where consolidation durvalumab after chemoradiotherapy (PACIFIC) significantly improved survival [19], while concurrent durvalumab with chemoradiotherapy (PACIFIC-2) failed to demonstrate benefit and was associated with greater toxicity [20].

To date, no systematic review or meta-analysis has directly compared concurrent versus sequential ICI timing in the neoadjuvant treatment of resectable ESCC. Existing reviews have largely pooled these schedules together or focused on ICI-versus-no-ICI comparisons, obscuring differences potentially attributable to scheduling. This gap carries direct clinical implications for treatment planning, surgical decision-making, and the design of future randomized trials. The present systematic review and meta-analysis therefore aims to synthesize all available evidence comparing the efficacy and safety of concurrent versus sequential ICI administration in the neoadjuvant treatment of patients with non-metastatic, resectable ESCC.

## 2 Methods

This systematic review and meta-analysis was conducted and reported in accordance with the Preferred Reporting Items for Systematic Reviews and Meta-Analyses (PRISMA) 2020 guidelines. The review protocol was registered prospectively in the International Prospective Register of Systematic Reviews (PROSPERO; registration number CRD420261422460) on 12 June 2026. The protocol registration was completed after the literature search and full-text screening had been finalized but before data extraction was initiated.

### 2.1 Information sources and search strategy

Three electronic databases were searched including PubMed, Scopus, and Embase covering all records from inception through April 2nd, 2026. Search terms were built around two conceptual domains: the disease and treatment setting (esophageal squamous cell carcinoma, neoadjuvant chemotherapy, radiotherapy, or chemoradiotherapy) and the intervention of interest (immune checkpoint inhibitors, PD-1 inhibitors, PD-L1 inhibitors, CTLA-4 inhibitors, immunotherapy). The detailed search strategies for all databases, including the complete search terms and search syntax, are provided in the Supplementary Material. Both controlled vocabulary terms and free-text keywords were used across all databases. In addition, reference lists of included articles and pertinent review papers were hand-searched to capture studies that may have been missed electronically.

### 2.2 Eligibility criteria

The PICOS framework guided the definition of eligibility criteria. The target population was adult patients (≥18 years) with histologically confirmed, non-metastatic, resectable ESCC. Eligible studies had to evaluate ICI therapy of any class delivered alongside neoadjuvant chemotherapy or chemoradiotherapy, with the central question being whether the timing of ICI administration relative to cytotoxic therapy influenced outcomes. Only prospective studies were considered, including both interventional clinical trials of any phase and prospective observational cohorts. At least one of the prespecified outcomes had to be reported for a study to qualify.

Studies were classified according to the timing of ICI administration relative to neoadjuvant chemotherapy or chemoradiotherapy (CRT). Concurrent ICI was defined as administration of ICI during the cytotoxic treatment phase, such that ICI was given concomitantly with chemotherapy or CRT for at least part of the neoadjuvant course. This category included regimens in which ICI was initiated during the concurrent chemotherapy or CRT phase; however, regimens in which ICI was administered both during an induction phase and subsequently continued concurrently with chemotherapy or CRT were considered a separate induction plus concurrent ICI regimen and were not grouped under concurrent ICI. Consolidation ICI was defined as administration of ICI exclusively after completion of neoadjuvant chemotherapy or CRT, with the first ICI dose given after the cytotoxic treatment had ended and before surgery.

Studies were excluded if they enrolled patients with metastatic or unresectable disease, or if treatment intent was definitive rather than neoadjuvant. Retrospective designs were not eligible. Other exclusions included case reports, review articles, editorials, conference abstracts lacking relevant data, preclinical work, and non-English publications. When the same patient cohort appeared across multiple publications, only the report providing the most complete outcome data was included.

### 2.3 Study selection

Two reviewers screened titles and abstracts independently, followed by full-text review of all records meeting initial criteria. Disagreements were settled by discussion, and a third reviewer was consulted when consensus was not reached. The full selection process is depicted in a PRISMA flow diagram (Supplementary Fig. 1).

### 2.4 Data extraction

A pre-designed data extraction form was used to systematically collect study-level, patient-level, treatment, and outcome data. One reviewer performed the primary data extraction, and a second reviewer independently cross-checked all extracted data for accuracy and completeness. Any discrepancies between the reviewers were resolved through discussion and consensus, with consultation with a third reviewer when necessary. Extracted information included study characteristics (study design, publication year, country, and sample size), patient and disease characteristics (age, sex, and clinical stage), treatment details (ICI agent, timing and sequence of ICI administration, chemotherapy backbone, and radiotherapy parameters), and all relevant outcome data. The primary outcome was pathological complete response (pCR), while secondary outcomes included major pathological response (MPR), overall survival (OS), disease-free survival (DFS), and treatment-related adverse events. When multiple publications reported data from the same study cohort, the report providing the most complete or recent data was used to avoid double counting. Where outcome data were presented only in figures, numerical values were estimated using validated digital extraction tools.

Pathological complete response (pCR) was defined as no evidence of residual tumor cells. When studies reported both pCR in the primary tumor alone and pCR in the primary tumor and regional lymph nodes, the latter definition was preferentially adopted. When the study did not explicitly clarify whether pCR assessment included regional lymph nodes, pCR was considered to encompass both the primary tumor and regional lymph nodes. Major pathological response (MPR) was defined as the presence of ≤10% residual viable tumor cells in the resected primary tumor bed.

### 2.5 Risk of bias assessment

Two reviewers independently appraised the methodological quality of each included study. ROBINS-I tool was used assessing risk of bias. Discrepancies were resolved by discussion or by involving a third reviewer.

### 2.6 Statistical analysis

All analyses were run in R (version 4.5.1). Because meaningful clinical and methodological diversity was anticipated across studies, stemming from differences in ICI agent, timing sequence, chemotherapy regimen, and radiotherapy use, random-effects models were chosen as the primary analytic approach. Pooled proportions were computed for single-arm data and back-transformed prior to reporting. Statistical significance was defined as a two-sided *p*-value below 0.05.

Heterogeneity was evaluated using Cochran's Q test alongside the I^2^ statistic and tau-squared. Values of I^2^ above 50% or a significant Q test were taken as evidence of substantial heterogeneity. Where the number of studies permitted, subgroup analyses and meta-regression were used to investigate potential drivers of heterogeneity. Publication bias was examined through funnel plot inspection and Egger's test, with the latter interpreted cautiously in the context of a potentially small study pool. Leave-one-out sensitivity analyses and comparisons between fixed- and random-effects models were performed to test the stability of all pooled results.

## 3 Results

### 3.1 Study selection

A total of 2853 records were retrieved from PubMed, Embase, and Scopus. Following the removal of 1113 duplicate records, 1740 titles and abstracts were screened, of which 1416 were excluded for irrelevance. The remaining 324 articles underwent full-text review, and ultimately 53 studies satisfied all inclusion criteria and were carried forward into the systematic review and meta-analysis [12], [21], [22], [23], [24], [25], [26], [27], [28], [29], [30], [31], [32], [33], [34], [35], [36], [37], [38], [39], [40], [41], [42], [43], [44], [45], [46], [47], [48], [49], [50], [51], [52], [53], [54], [55], [56], [57], [58], [59], [60], [61], [62], [63], [64], [65], [66], [67], [68], [69], [70], [71], [72]. The selection process is illustrated in the PRISMA flow diagram (Supplementary Fig. 1).

Potentially overlapping patient cohorts were assessed by comparing clinical trial registration numbers, author lists, institutional affiliations, study periods, patient populations, treatment protocols, ethics approval numbers, and cross-references between publications. When overlapping reports were identified, the most recent publication was used as the primary source for outcome extraction, while outcomes available only in earlier reports were additionally extracted without duplicating participants. This approach minimized the risk of double-counting while ensuring comprehensive data collection.

### 3.2 Included studies and patients

Overall, 53 prospective studies met the inclusion criteria. A total of 2937 patients with non-metastatic resectable ESCC received neoadjuvant therapy with concurrent or sequential ICI, among whom a total of 2401 underwent surgery. Among those who underwent esophagectomy, 2238 patients in 48 studies received concurrent ICI in their neoadjuvant regimen, while 108 patients received consolidation ICI in 4 studies, and 55 patients received induction and concurrent ICI in only 1 study.

Study characteristics are summarized in Supplementary Table 1. The studies were published between 2021 and 2026. All of the studies were conducted in China, except for two, which were conducted in Taiwan and Korea. Patient age ranged from 31 to 79 years, and the proportion of male patients across reporting studies ranged from 60% to 97.3%.

All included studies used PD-1 inhibitors as the ICI agent. Sintilimab, camrelizumab, toripalimab, and tislelizumab were the most commonly used agents, with pembrolizumab appearing in a small number of studies. No study employed a PD-L1 or CTLA-4 inhibitor. Chemotherapy backbones consisted most commonly of a taxane (paclitaxel in its conventional, liposomal, or albumin-bound formulation) paired with either carboplatin or cisplatin. A minority of studies used docetaxel or capecitabine-based regimens. Neoadjuvant chemoradiotherapy was used in 14 studies, while chemotherapy or radiotherapy alone was used in 39 studies. ICI timing relative to cytotoxic therapy differed across studies, with 48 studies employing a concurrent approach and 5 adopting a sequential strategy, with 4 consolidation studies and 1 induction plus concurrent study.

### 3.3 Risk of bias assessment

According to the ROBINS-I assessment, 9 studies were judged to have an overall serious risk of bias, with the majority of these judgments primarily driven by the missing data (D5) domain. An additional 11 studies were considered to have a moderate risk of bias, while the remaining studies were classified as having a low risk of bias. Individual domain-specific and overall risk-of-bias assessments are presented in Supplementary Fig. 2.

### 3.4 Pathological complete response: concurrent vs. consolidation ICI

Since only one study regarding induction ICI was detected, pCR was compared between concurrent and consolidation timings. pCR rates were compared between patients receiving concurrent ICI and those receiving consolidation ICI across 52 studies comprising a total of 2238 patients in the concurrent arm and 108 patients in the consolidation arm. The pooled analysis demonstrated a significant difference in pCR rate between the two groups (48% [95%CI: 39%–58%] vs. 31% [95%CI: 28%–34%] for consolidation vs. concurrent ICI, *p* = 0.0005).

However, since all the studies in the sequential subgroup used ICI in combination with chemoradiotherapy and many of the studies in the concurrent subgroup used immunochemotherapy or immunoradiotherapy, additionally, we decided to compare the pCR rates between concurrent and consolidation ICI, only using the studies that combined it with chemoradiotherapy. This time, despite being numerically higher again, the pCR rate in the consolidation group did not demonstrate a statistically significant superiority compared to the consolidation group (48% [95%CI: 39%–58%] vs. 41% [95%CI: 36%–46%], for consolidation vs. concurrent ICI, *p* = 0.1797). This indicate that a big proportion of the superiority of consolidation in the first analysis might have been due to the usage of combined chemotherapy and radiotherapy, rather than the ICI timing.

**Fig. 1:**
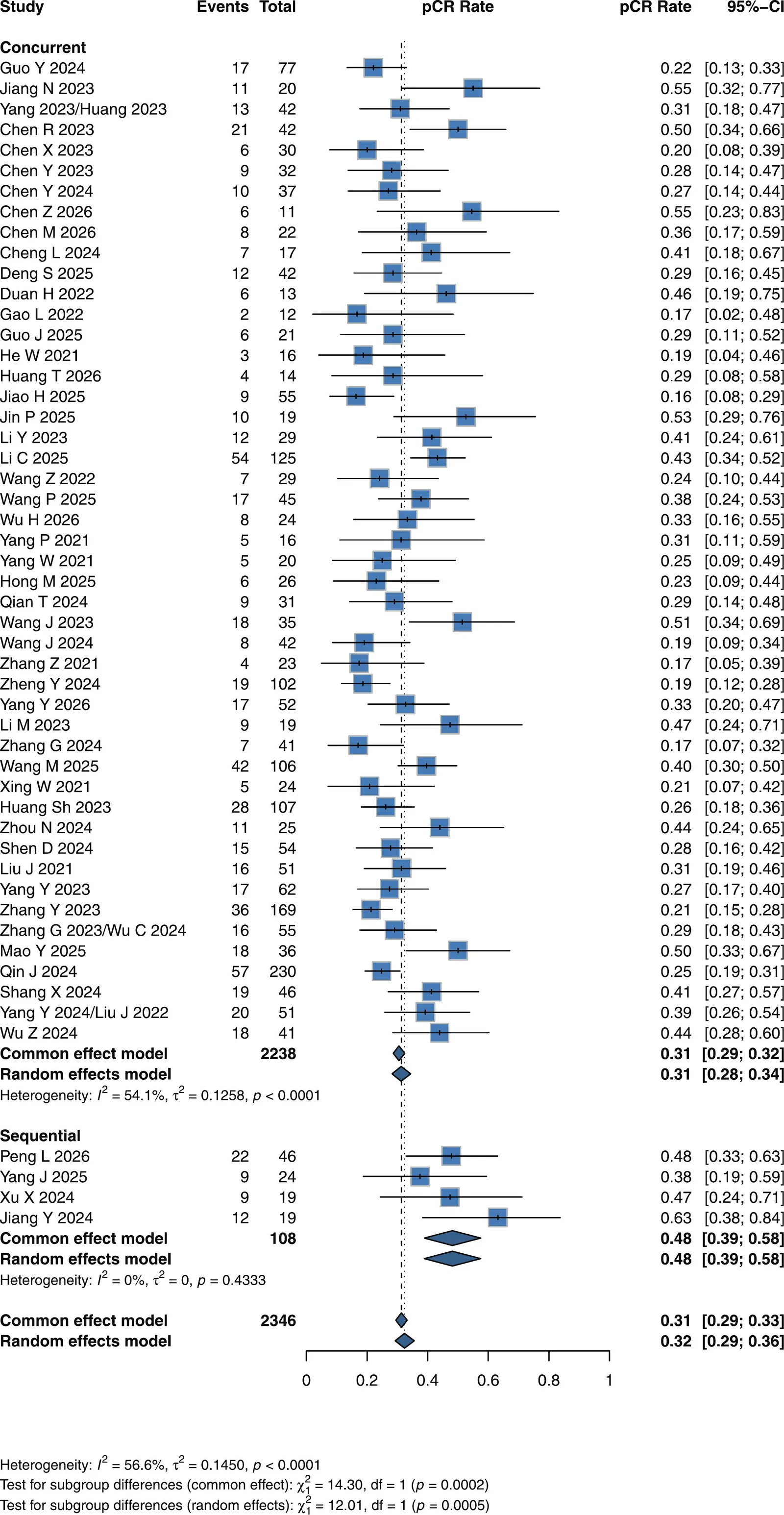

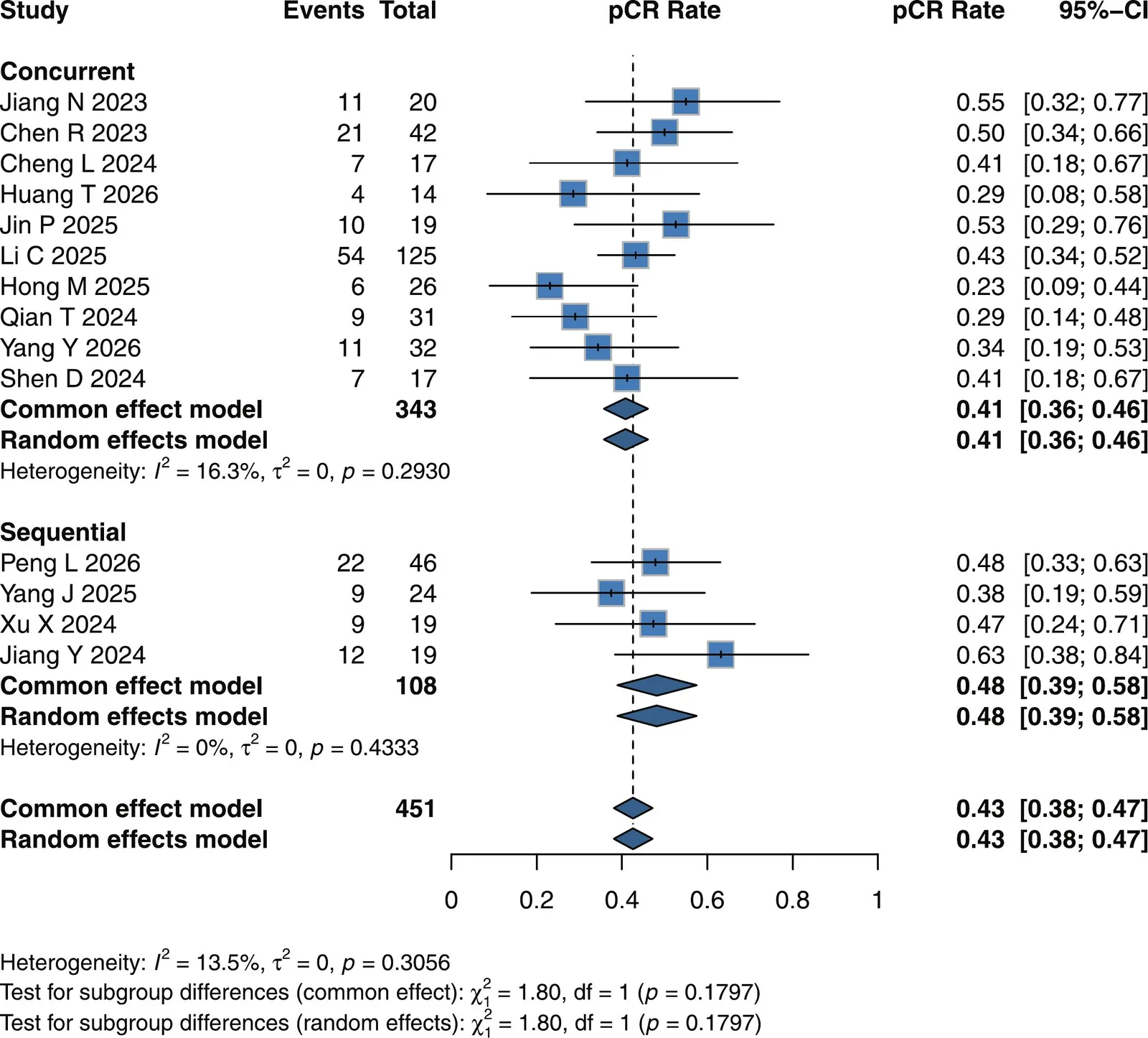
Forest plots demonstrating the comparison of concurrent and consolidation ICI regarding pathological complete response (pCR), A. All studies B. neoadjuvant immunochemotherapy-only studies.

Between-study heterogeneity was low for the concurrent neoadjuvant ICRT (I^2^ = 16.3%, τ^2^ = 0, *p* = 0.2930) and consolidation neoadjuvant ICRT (I^2^ = 0%, τ^2^ = 0, *p* = 0.4333). Egger's test did not suggest significant publication bias among concurrent studies (*p* = 0.6669); however, this finding should be interpreted cautiously given the small number of studies available for assessment. The corresponding forest plots are displayed in Fig. 1.

### 3.5 Major pathological response: concurrent vs. sequential ICI

MPR, defined as 10% or less residual viable tumor in the surgical specimen, was assessable in 41 studies including 1724 patients. Again, the pooled comparison between concurrent and consolidation ICI groups yielded a significantly higher MPR rate in the consolidation group at first (76% (95%CI: 62%–86%) for consolidation vs. 57% (95%CI: 52%–61%); *p* = 0.0116), but the difference became statistically insignificant as we only brought the concurrent studies that used chemoradiotherapy (76% (95%CI: 62%–86%) for consolidation vs. 70% (95%CI: 61%–78%); *p* = 0.2702).

Between-study heterogeneity was high for the concurrent neoadjuvant ICRT (I^2^ = 73.8%, τ^2^ = 0.2730, *p* = 0.0019) and the consolidation neoadjuvant ICRT (I^2^ = 52.3%, τ^2^ = 0.1686, *p* = 0.0984). Although differences in treatment characteristics across studies, including variations in the neoadjuvant treatment backbone and regimen, may have contributed to the observed heterogeneity, formal meta-regression or additional subgroup analyses were not performed because of the small number of studies within each subgroup. Such analyses would have had limited statistical power and could have yielded unstable or potentially misleading estimates. Importantly, the primary objective of this study was to compare pathological response rates between concurrent and consolidation ICI strategies rather than to identify clinical determinants of heterogeneity within each treatment approach; therefore, a detailed exploration of heterogeneity sources was beyond the scope of this analysis. As a result, the exact sources of heterogeneity remain unclear, and the pooled estimates should be interpreted in light of the observed between-study variability.

**Fig. 2:**
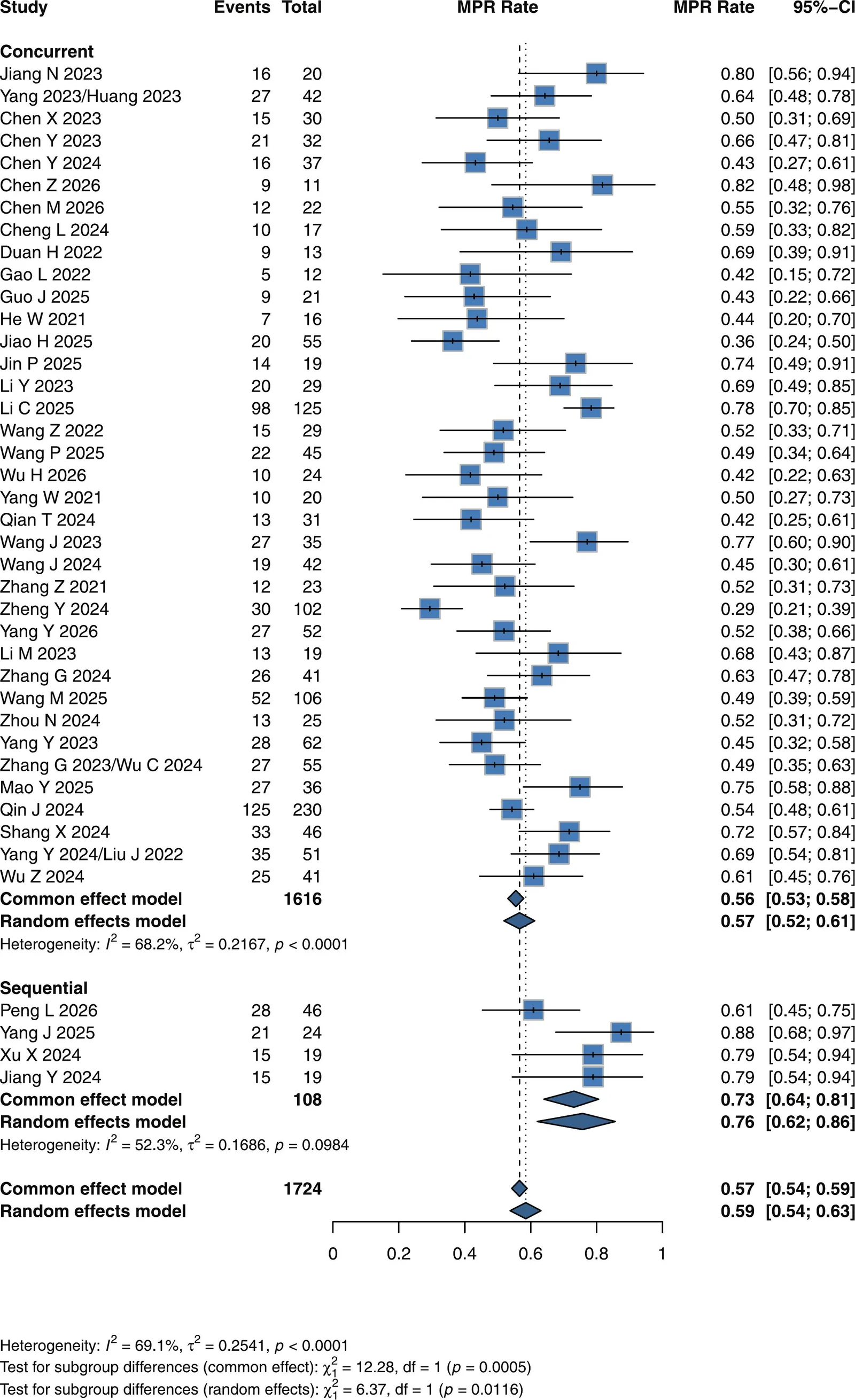

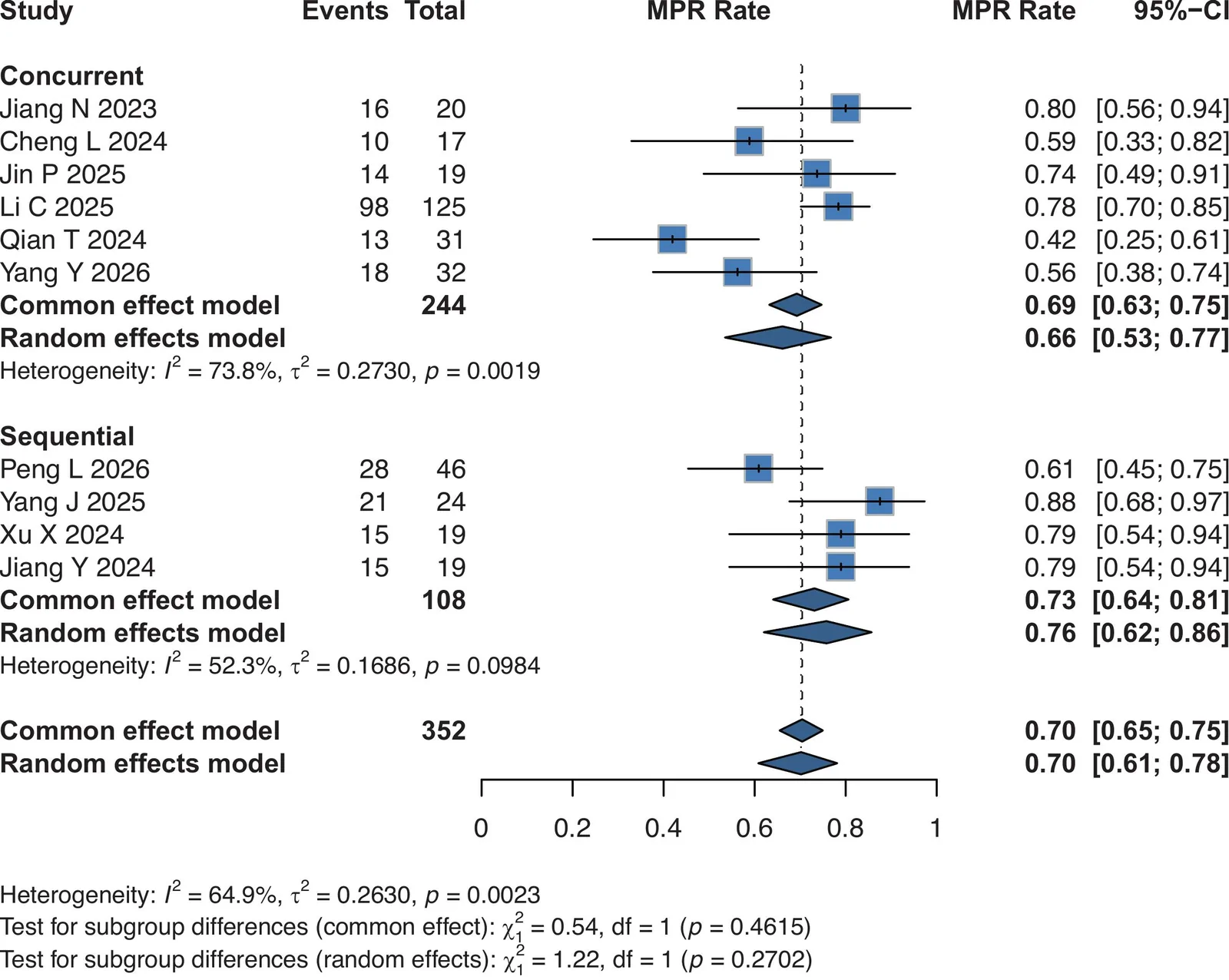
Forest plots demonstrating the comparison of concurrent and consolidation ICI regarding major pathological response (MPR), A. All studies B. neoadjuvant immunochemotherapy-only studies.

Egger's test did not suggest significant publication bias among concurrent studies (*p* = 0.6669); however, this finding should be interpreted cautiously given the small number of studies available for assessment. The corresponding forest plots are displayed in Fig. 2.

## 4 Discussion

This systematic review and meta-analysis is, to our knowledge, the first to directly compare the efficacy and safety of concurrent versus consolidation ICI administration in the neoadjuvant treatment of resectable ESCC. Our primary analysis showed that consolidation ICI was associated with a numerically and statistically higher pCR rate compared to concurrent ICI when all studies were pooled together. However, this advantage lost statistical significance once the comparison was restricted to studies that used chemoradiotherapy in both arms, with pCR rates of 48% versus 41% for consolidation and concurrent ICI, respectively. A similar pattern was observed for MPR. These findings suggest that the apparent superiority of consolidation ICI in the primary analysis was driven largely by the greater use of chemoradiotherapy in the sequential subgroup, rather than by the timing of ICI delivery itself. Of note, a recent meta-analysis found that ICRT significantly improved pCR rates compared to ICT [14]. When consolidation studies were disproportionately enriched with chemoradiotherapy regimens, any raw between-group comparison was inherently confounded by the modality difference. Our sensitivity analysis correcting for this imbalance effectively neutralized the timing-related signal. Nevertheless, these findings should be interpreted cautiously given the relatively small consolidation subgroup and the predominance of non-randomized, single-arm studies, which limit the ability to establish comparative effectiveness or draw causal inferences regarding ICI timing. Therefore, the observed differences should be considered hypothesis-generating rather than evidence of superiority of either treatment sequence.

From a mechanistic standpoint, both timing strategies carry theoretical rationale. As mentioned earlier, concurrent ICI may leverage radiation-induced immunogenic cell death and the release of DAMPs to prime the immune system at a moment when antigen presentation is most active [15]. Upregulation of PD-L1 by radiotherapy within the tumor microenvironment further supports the concurrent approach by creating a more permissive context for checkpoint blockade [15]. However, this benefit is counterbalanced by the well-documented phenomenon of radiation-induced lymphopenia, a direct consequence of the high radiosensitivity of circulating lymphocytes even at low radiation doses [16]. Since ICI efficacy depends on an intact pool of effector T cells, severe lymphopenia may blunt the immunological response precisely when checkpoint inhibition is applied [16].

The consolidation strategy, by administering ICI after the cytotoxic phase theoretically avoids this interference, allowing lymphocyte recovery before immune engagement [73]. It is uncertain whether or not lymphocyte recovery after chemoradiotherapy is sufficient to meaningfully restore immune competence before ICI administration. However, in a dedicated analysis of ESCC patients undergoing definitive concurrent chemoradiotherapy, only 44.8% had lymphocyte counts return to normal levels at six months post-treatment, with the majority still experiencing residual lymphopenia of varying severity [74]. More critically, lymphocyte recovery did not rescue the prognostic penalty of severe lymphopenia, as patients who had experienced grade 4 lymphopenia during treatment continued to have significantly worse overall survival even after apparent count recovery, suggesting that numerical reconstitution does not fully reflect functional immune restoration [75]. This raises the possibility that the consolidation strategy's theoretical immunological advantage may be attenuated in patients who sustain deep lymphocyte nadirs during the cytotoxic phase, and that the quality rather than simply the quantity of recovering lymphocytes determines whether ICI engagement is ultimately effective [76].

While these immunological mechanisms provide a plausible rationale for the consolidation strategy, they should be interpreted within the biological context of ESCC, as important histology-specific differences exist between ESCC and esophageal adenocarcinoma (EAC). Compared with EAC, ESCC demonstrates greater sensitivity to both radiotherapy and immune checkpoint inhibition. Evidence from the CROSS trial showed that the addition of neoadjuvant chemoradiotherapy resulted in substantially greater survival and pathological response benefits in patients with ESCC than in those with EAC, with pathological complete response rates of 49% and 23%, respectively, underscoring the greater radiosensitivity of squamous histology [3], [4]. Likewise, a meta-analysis of phase III randomized trials found that immune checkpoint inhibitors significantly improved overall survival in patients with ESCC but not in those with EAC [77]. These differences are supported by distinct tumor biology. ESCC is characterized by a more immunogenic tumor microenvironment, including a higher tumor mutational burden, greater infiltration of immune-effector cells, lower levels of regulatory T cells, and differential immune checkpoint gene expression, all of which are associated with enhanced responsiveness to immune checkpoint blockade. In contrast, EAC exhibits a relatively immunosuppressive microenvironment with fewer neoantigens and predominant regulatory T-cell infiltration, contributing to reduced sensitivity to immunotherapy [77]. Therefore, the potential interaction between radiotherapy and immunotherapy, as well as the influence of the treatment backbone observed in our meta-analysis, should be interpreted as being specific to ESCC and should not be directly extrapolated to EAC.

However, there are several limitations regarding our study that merit careful consideration. First, the evidence base for sequential ICI timing remains sparse. Only four consolidation studies contributed to the comparative analyses, and two of these were published only as conference abstracts without full peer-reviewed data. This substantially limits the precision of estimates for the sequential subgroup and introduces a risk of incomplete outcome reporting. Although the pooled results were directionally consistent, the consolidation arm was insufficiently powered for definitive conclusions, and the findings should be interpreted with appropriate caution until more mature data become available. Second, the generalizability of our findings is limited by the geographic and therapeutic characteristics of the available evidence. Almost all included studies were conducted in China, which may limit the applicability of the findings to other ethnic populations and healthcare settings with differences in nutritional status, comorbidity profiles, and surgical volumes. In addition, all included studies evaluated PD-1 inhibitors; therefore, whether these findings apply to other classes of ICIs remains uncertain. Third, despite our sensitivity analyses, residual confounding from differences in chemotherapy regimens, radiation doses and target volumes, and fractionation schedules across studies cannot be fully excluded. Fourth, we were only able to find one study in which patients were administered with induction ICI therapy in the neoadjuvant setting, meaning that we were not able to analyze this group of patients and compare their outcomes with other ICI timing strategies. Fifth, quantitative synthesis was not performed for the secondary outcomes of treatment-related adverse events, disease-free survival (DFS), and overall survival (OS). This decision was based on the limited availability of comparable data, particularly within the consolidation ICI group. Only two consolidation studies reported OS and DFS [70], [71], precluding meaningful pooled comparisons between treatment strategies. Likewise, safety outcomes were reported inconsistently across studies, with substantial heterogeneity in adverse event definitions, grading, and reporting formats. Finally, the substantial heterogeneity observed in the concurrent ICI secondary analysis of MPR (I^2^ = 73.8%) could not be further explored using meta-regression, as this was beyond the scope of the present study and the number of available studies was limited; therefore, the clinical and methodological factors underlying this variability remain uncertain.

Despite these limitations, this review makes a meaningful contribution by being the first to systematically isolate the effect of ICI timing in the neoadjuvant ESCC setting, and by demonstrating that the choice between concurrent and consolidation administration, when matched for backbone regimen, does not appear to produce meaningfully different pathological response rates based on current evidence. This has practical implications, as it may provide reassurance that consolidation scheduling is not inferior to concurrent delivery, and it opens the door to designing schedules that prioritize immune preservation by avoiding the lymphopenic nadir of chemoradiotherapy. Notably, these conclusions align closely with the recently published HCHTOG1906 trial, the first randomized comparison of sequential versus concurrent neoadjuvant immunochemotherapy in ESCC, which found no significant difference in pCR between the two schedules but reported a higher incidence of fatal immune-related adverse events in the concurrent arm [78].

## 5 Conclusion

In summary, the available evidence suggests that the timing of ICI administration relative to chemoradiotherapy may not independently determine pathological response in resectable ESCC; however, these findings are hypothesis-generating and should be interpreted cautiously given the observational nature and heterogeneity of the available evidence. Prospective randomized trials with pre-specified timing arms and stratification by treatment backbone are warranted to determine the optimal sequencing of ICI therapy in this evolving field.

## CRediT authorship contribution statement

**Reza Ghalehtaki:** Writing – review & editing, Conceptualization. **Alireza Shariati:** Writing – review & editing, Writing – original draft, Formal analysis, Conceptualization. **Taher Fanihagh:** Methodology, Investigation. **Zahra Moradi:** Methodology, Investigation. **Niloofar Moradi:** Methodology, Investigation.

## Funding

This study received no specific funding from any public, commercial, or not-for-profit funding agency.

## Declaration of generative AI and AI-assisted technologies in the manuscript preparation process

During the preparation of this manuscript, the authors used ChatGPT for drafting assistance and language refinement. The authors critically reviewed and revised all AI-assisted content and take full responsibility for the accuracy, originality, and integrity of the final manuscript. No AI tool was used to generate or fabricate scientific data, results, or references.

## Declaration of competing interest

The authors declare that they have no known competing financial interests or personal relationships that could have appeared to influence the work reported in this paper.

## Appendix A Supplementary data

Supplementary material 1 Search strategy
