# Supplementary material for: Concurrent versus sequential immune checkpoint inhibition in the neoadjuvant treatment of resectable esophageal squamous cell carcinoma: a systematic review and meta-analysis
Source: Clin Transl Radiat Oncol. 2026 Sep 10;62:101289. doi: 10.1016/j.ctro.2026.101289 (PMC13601065; doi:10.1016/j.ctro.2026.101289)
Supplement: Supplementary file 1 [file mmc1.docx]

Search strategy for electronic databases:

April 2^nd^ 2026

Pubmed:

("Esophageal Squamous Cell Carcinoma"[Mesh] OR ("Esophageal Neoplasms"[Mesh] AND "Carcinoma, Squamous Cell"[Mesh]) OR (("esophag*"[tiab] OR "oesophag*"[tiab]) AND "squamous cell carcinoma"[tiab]) OR (("esophag*"[tiab] OR "oesophag*"[tiab]) AND "SCC"[tiab])))

AND

("Immune Checkpoint Inhibitors"[Mesh] OR "immune checkpoint inhibitor*"[tiab] OR "Immunotherap*"[tiab] OR "ICI*"[tiab] OR "immune checkpoint block*"[tiab] OR "PD-1"[tiab] OR "PD L1"[tiab] OR "PD-L1"[tiab] OR "CD152"[tiab] OR "programmed death-ligand 1"[tiab] OR "CTLA-4"[tiab] OR "CTLA 4"[tiab] OR "camrelizumab"[tiab] OR "nivolumab"[tiab] OR "pembrolizumab"[tiab] OR "tremelimumab"[tiab] OR "atezolizumab"[tiab] OR "toripalimab"[tiab] OR "cemiplimab"[tiab] OR "tislelizumab"[tiab] OR "avelumab"[tiab] OR "durvalumab"[tiab] OR "ipilimumab"[tiab] OR "sintilimab"[tiab])

AND

("Neoadjuvant Therapy"[Mesh] OR "neoadjuvant"[tiab] OR "preoperative"[tiab] OR "pre-operative"[tiab] OR "resectable"[tiab] OR "resection"[tiab] OR "resected"[tiab] OR "operable"[tiab] OR "esophagectomy"[tiab] OR "locally advanced"[tiab])

727 results

Scopus:

(TITLE-ABS-KEY(("esophagus" OR "oesophagus" OR "esophageal" OR "oesophageal") AND ("squamous cell carcinoma" OR "SCC")))

AND

(TITLE-ABS-KEY("immune checkpoint inhibitor" OR "ICI" OR "immune checkpoint blockade" OR "PD-1" OR "PD L1" OR "PD-L1" OR "CD152" OR "programmed death-ligand 1" OR "CTLA-4" OR "CTLA 4" OR "camrelizumab" OR "nivolumab" OR "pembrolizumab" OR "tremelimumab" OR "atezolizumab" OR "toripalimab" OR "cemiplimab" OR "tislelizumab" OR "avelumab" OR "durvalumab" OR "ipilimumab" OR "sintilimab"))

AND

(TITLE-ABS-KEY("neoadjuvant" OR "preoperative" OR "pre-operative" OR "resectable" OR "resection" OR "resected" OR "operable" OR "esophagectomy" OR "locally advanced"))

965 results

Embase:

('esophageal squamous cell carcinoma'/exp OR (esophag*:ti,ab OR oesophag*:ti,ab) AND ('squamous cell carcinoma':ti,ab OR SCC:ti,ab))

AND

('neoadjuvant':ti,ab,kw OR 'preoperative':ti,ab,kw OR 'pre-operative':ti,ab,kw OR 'resectable':ti,ab,kw OR 'resection':ti,ab,kw OR 'resected':ti,ab,kw OR 'operable':ti,ab,kw OR 'esophagectomy':ti,ab,kw OR "locally advanced":ti,ab,kw)

AND

('immune checkpoint inhibitor'/exp OR 'immune checkpoint inhibitor*':ti,ab OR ICI*:ti,ab OR 'immune checkpoint block*':ti,ab OR 'PD-1':ti,ab OR 'PD L1':ti,ab OR 'PD-L1':ti,ab OR CD152:ti,ab OR 'programmed death-ligand 1':ti,ab OR CTLA-4:ti,ab OR 'CTLA 4':ti,ab OR camrelizumab:ti,ab OR nivolumab:ti,ab OR pembrolizumab:ti,ab OR tremelimumab:ti,ab OR atezolizumab:ti,ab OR toripalimab:ti,ab OR cemiplimab:ti,ab OR tislelizumab:ti,ab OR avelumab:ti,ab OR durvalumab:ti,ab OR ipilimumab:ti,ab OR sintilimab:ti,ab)

1161 results
