# Supplementary material for: Concurrent versus sequential immune checkpoint inhibition in the neoadjuvant treatment of resectable esophageal squamous cell carcinoma: a systematic review and meta-analysis
Source: Clin Transl Radiat Oncol. 2026 Sep 10;62:101289. doi: 10.1016/j.ctro.2026.101289 (PMC13601065; doi:10.1016/j.ctro.2026.101289)
Supplement: Supplementary file 2 [file mmc2.docx]

Figure 1. PRISMA flowchart

Figure 2. Risk of bias assessment using ROBINS-I tool.
